# Supplementary material for: STAG2 regulates interferon signaling in melanoma via enhancer loop reprogramming
Source: Nat Commun. 2022 Apr 6;13:1859. doi: 10.1038/s41467-022-29541-9 (PMC8986786; doi:10.1038/s41467-022-29541-9)
Supplement: Supplementary file 1 — Supplementary Information [file 41467_2022_29541_MOESM1_ESM.pdf]

SUPPLEMENTARY INFORMATION

STAG2 regulates interferon signaling in melanoma via enhancer loop reprogramming

Zhaowei Chu[1,2,16], Lei Gu[3,16,*], Yeguang Hu[1,16], Xiaoyang Zhang[4,16], Man Li[1], Jiajia Chen[5,6], Da Teng[1], Man Huang[7], Che-Hung Shen[8], Li Cai[9], Toshimi Yoshida[1], Yifeng Qi[10], Zhixin Niu[3], Austin Feng[1], Songmei Geng[11], Dennie T. Frederick[12], Emma Specht[12], Adriano Piris[13], Ryan J. Sullivan[12], Keith T. Flaherty[12], Genevieve M. Boland[14], Katia Georgopoulos[1], David Liu[5,6], Yang Shi[7,15], Bin Zheng[1,*]

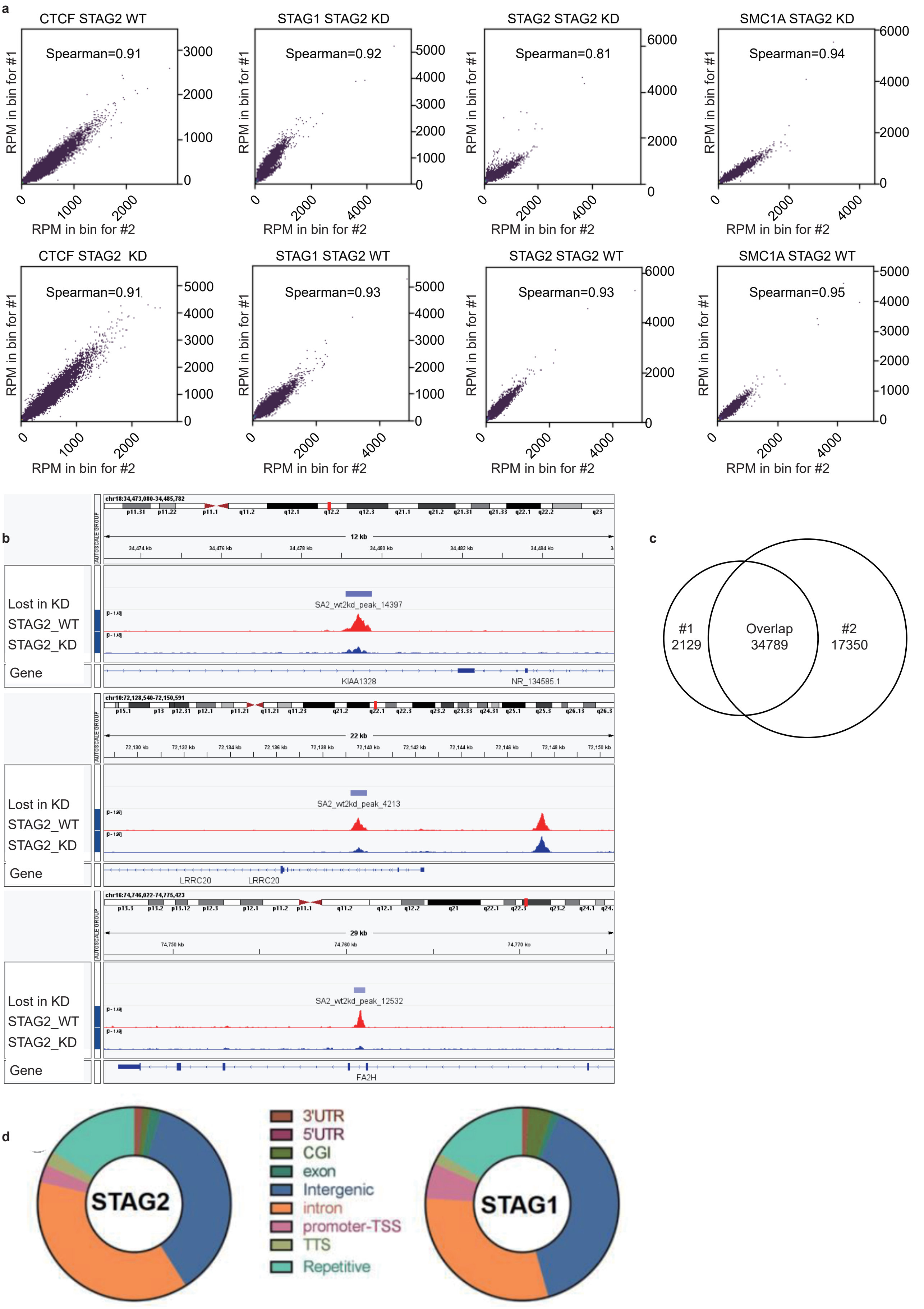
a
CTCF STAG2 WT
Spearman=0.91
STAG1 STAG2 KD
Spearman=0.92
STAG2 STAG2 KD
Spearman=0.81
SMC1A STAG2 KD
Spearman=0.94
CTCF STAG2 KD
Spearman=0.91
STAG1 STAG2 WT
Spearman=0.93
STAG2 STAG2 WT
Spearman=0.93
SMC1A STAG2 WT
Spearman=0.95
RPM in bin for #1
RPM in bin for #2
b
Lost in KD
STAG2_WT
STAG2_KD
Gene
SA2_wt2kd_peak_14397
KIAA1328
NR_134585.1
SA2_wt2kd_peak_4213
LRRC20
LRRC20
SA2_wt2kd_peak_12532
FA2H
c
#1
2129
Overlap
34789
#2
17350
d
STAG2
STAG1
3'UTR
5'UTR
CGI
exon
Intergenic
intron
promoter-TSS
TTS
Repetitive

Supplementary Figure 1. **Characterization of STAG2 binding sites in M14 melanoma cells by ChIP-seq. a** High correlation of ChIP-seq data between biological replicates. High Spearman's correlation of the reads distribution from ChIP-seq experiments was observed between two biological replicates of M14 cells without (WT) or with (KD) STAG2 knockdown. Spearman correlation is calculated with reads per million (RPM) in each 10Kb window throughout the whole genome. **b** Examples of loss of STAG2 binding sites. Bigwig files for each sample is uploaded into IGV. Three representatives on loss of STAG2 binding sites are shown. **c** Overlap of STAG2 differential binding sites between two biological replicates. **d** The genomic distribution of differential binding sites for STAG1 and STAG2.

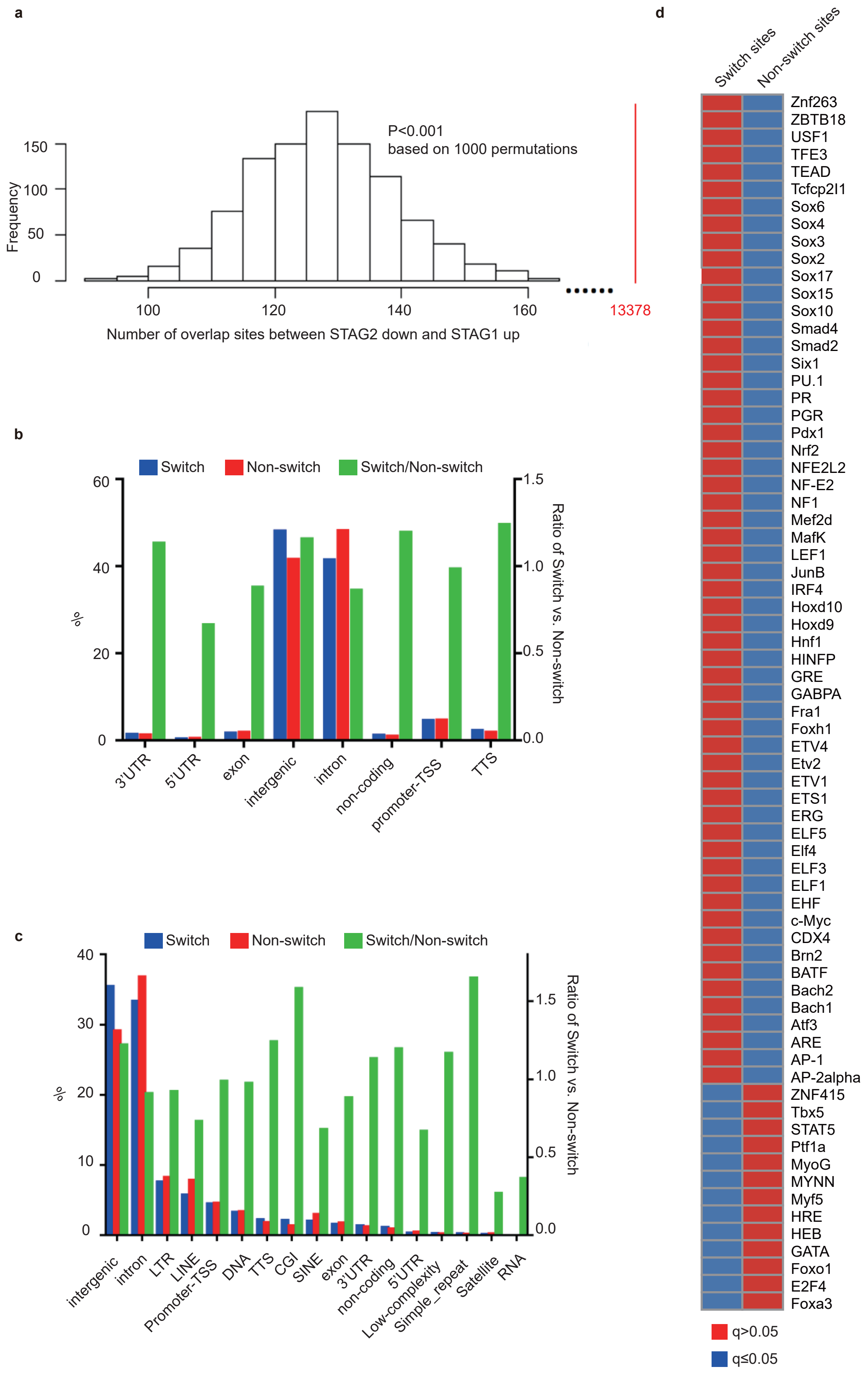
a
P<0.001
based on 1000 permutations
Frequency
150
100
50
0
100
120
140
160
13378
Number of overlap sites between STAG2 down and STAG1 up
b
Switch
Non-switch
Switch/Non-switch
%
60
40
20
0
1.5
1.0
0.5
0.0
Ratio of Switch vs. Non-switch
3'UTR
5'UTR
exon
intergenic
intron
non-coding
promoter-TSS
TTS
c
Switch
Non-switch
Switch/Non-switch
%
40
30
20
10
0
1.5
1.0
0.5
0.0
Ratio of Switch vs. Non-switch
intergenic
intron
LTR
LINE
Promoter-TSS
DNA
TTS
CGI
SINE
exon
3'UTR
non-coding
5'UTR
Low-complexity
Simple_repeat
Satellite
RNA
d
Switch sites
Non-switch sites
Znf263
ZBTB18
USF1
TFE3
TEAD
Tcfcp2l1
Sox6
Sox4
Sox3
Sox2
Sox17
Sox15
Sox10
Smad4
Smad2
Six1
PU.1
PR
PGR
Pdx1
Nrf2
NFE2L2
NF-E2
NF1
Mef2d
MafK
LEF1
JunB
IRF4
Hoxd10
Hoxd9
Hnf1
HINFP
GRE
GABPA
Fra1
Foxh1
ETV4
Etv2
ETV1
ETS1
ERG
ELF5
Elf4
ELF3
ELF1
EHF
c-Myc
CDX4
Brn2
BATF
Bach2
Bach1
Atf3
ARE
AP-1
AP-2alpha
ZNF415
Tbx5
STAT5
Ptf1a
MyoG
MYNN
Myf5
HRE
HEB
GATA
Foxo1
E2F4
Foxa3
q>0.05
q≤0.05

Supplementary Figure 2. Characterization of STAG2 to STAG1 switch sites and non-switch sites in M14 cells. a Distribution of overlap between randomly simulated differential STAG1 and STAG2 sites. Red bar is the observed overlap sites. Empirical P value is 0.001, and is calculated based on 1000 times permutation test. **b** General genomic distribution of switch and non-switch STAG2 sites. **c** Fine mapping of the switch and non-switch STAG2 sites. **d** Motif enriched at switched and non-switch STAG2 sites.

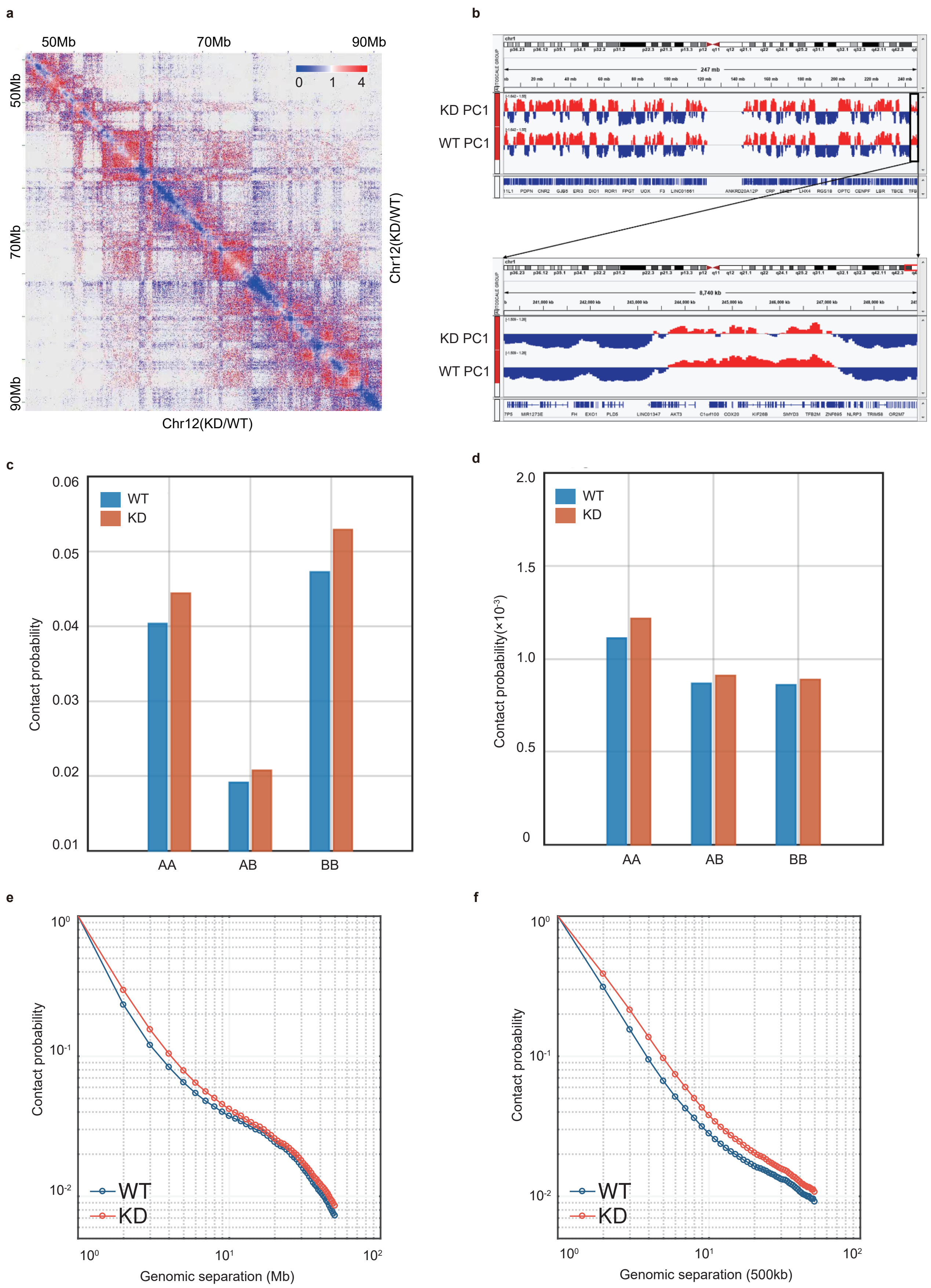
a
50Mb
70Mb
90Mb
0
1
4
Chr12(KD/WT)
Chr12(KD/WT)
b
KD PC1
WT PC1
KD PC1
WT PC1
chr1
247 mb
8,740 kb
c
WT
KD
Contact probability
0.06
0.05
0.04
0.03
0.02
0.01
AA
AB
BB
d
WT
KD
Contact probability(×10^{-3})
2.0
1.5
1.0
0.5
0
AA
AB
BB
e
Contact probability
10^0
10^{-1}
10^{-2}
WT
KD
10^0
10^1
10^2
Genomic separation (Mb)
f
Contact probability
10^0
10^{-1}
10^{-2}
WT
KD
10^0
10^1
10^2
Genomic separation (500kb)

Supplementary Figure 3. **STAG2 inactivation leads to reorganization of chromosomal folding. a** Representative heatmap for the global change of contact probability on chromosome 12 upon STAG2 knockdown in M14 cells. Reddish represents impaired contacts between genomic loci, and bluish represents enhanced contacts. **b** A representative example of fine-scale compartment changes between M14 cells without (WT) and with (KD) STAG2 knockdown. **c** Bar plot for average intra-chromosome contact probability within compartment A (AA) and B (BB), and across compartments (AB) in STAG2 WT and KD cells. **d** Bar plot for average inter-chromosome contact probability within compartment A (AA) and B (BB), and across compartments (AB) in STAG2 WT and KD cells. **e-f** Average contact probability at different genomic distance in STAG2 WT and KD cells, using 1Mb (**e**) or 500kb (**f**) or as window sizes.

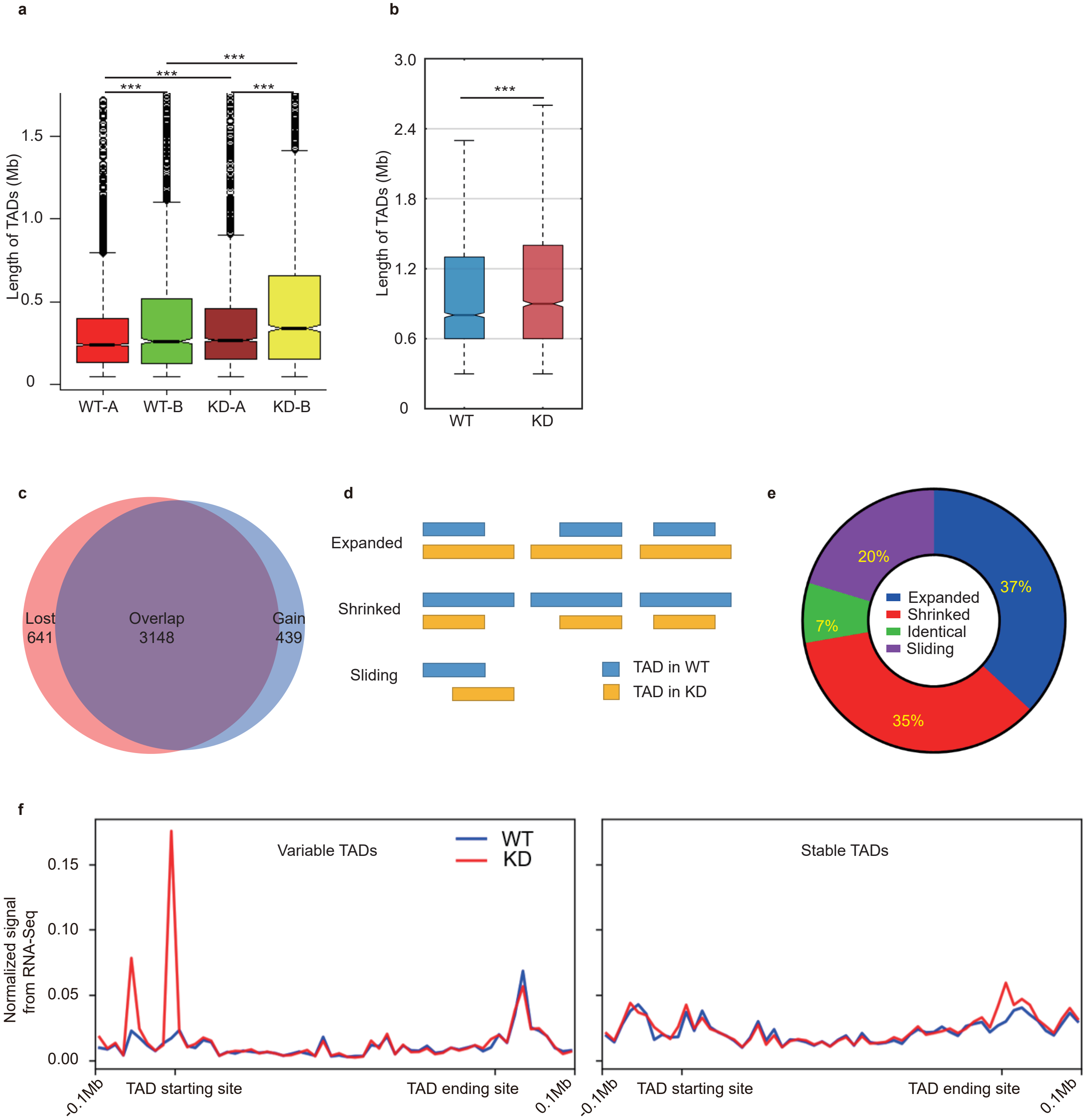
a
***
***
***
***
Length of TADs (Mb)
1.5
1.0
0.5
0
WT-A
WT-B
KD-A
KD-B
b
3.0
2.4
1.8
1.2
0.6
0
***
Length of TADs (Mb)
WT
KD
c
Lost
641
Overlap
3148
Gain
439
d
Expanded
Shrinked
Sliding
TAD in WT
TAD in KD
e
20%
37%
7%
35%
Expanded
Shrinked
Identical
Sliding
f
Variable TADs
WT
KD
Stable TADs
Normalized signal
from RNA-Seq
0.15
0.10
0.05
0.00
-0.1Mb
TAD starting site
TAD ending site
0.1Mb
-0.1Mb
TAD starting site
TAD ending site
0.1Mb

Supplementary Figure 4. STAG2 inactivation is associated with disruption of TAD boundaries. a Distribution of TAD length in A/B compartments in M14 cells without (WT) and with (KD) STAG2 knockdown (n= 1671, 2010, 1378, 2002 TADs in WT-A, WT-B, KD-A, KD-B, respectively). P value is 2.324e-15 (WT-A VS WT-B), 3.383e-17 (KD-A VS KD-B), 2.378e-13 (WT-A VS KD-A), 2.481e-13 (WT-B VS KD-B) and is based on two-sided *wilcox*-test. **b** TAD length is significantly different between STAG2 WT (n=3672 TADs) and KD (n=3423 TADs) cells, as cross-validated by TADbit. P value is 2.473e-11 and is based on two-sided *wilcox*-test. **c** Overview of changes in TADs upon STAG2 knockdown. Lost represents TADs despaired in STAG2 KD cells; gain represents newly formed TADs in STAG2 KD cells; overlap represents TADs showing up in both WT and KD cells. **d** Schematic representation of different types of TAD movements for overlap TADs upon knockdown of STAG2. **e** Percentages of different types of TAD movements within overlap TADs. **f** Meta plots of gene expression at boundaries of both variable TADs and stable TADs. Statistical significance is determined as: ns $P > 0.05$; *$P < 0.05$; **$P < 0.01$; ***$P < 0.001$. The box plot is defined by bounds at the 25th percentile and 75th percentile, centre at 50th percentile, the minima and maxima are at the 10th percentile and 90th percentile.

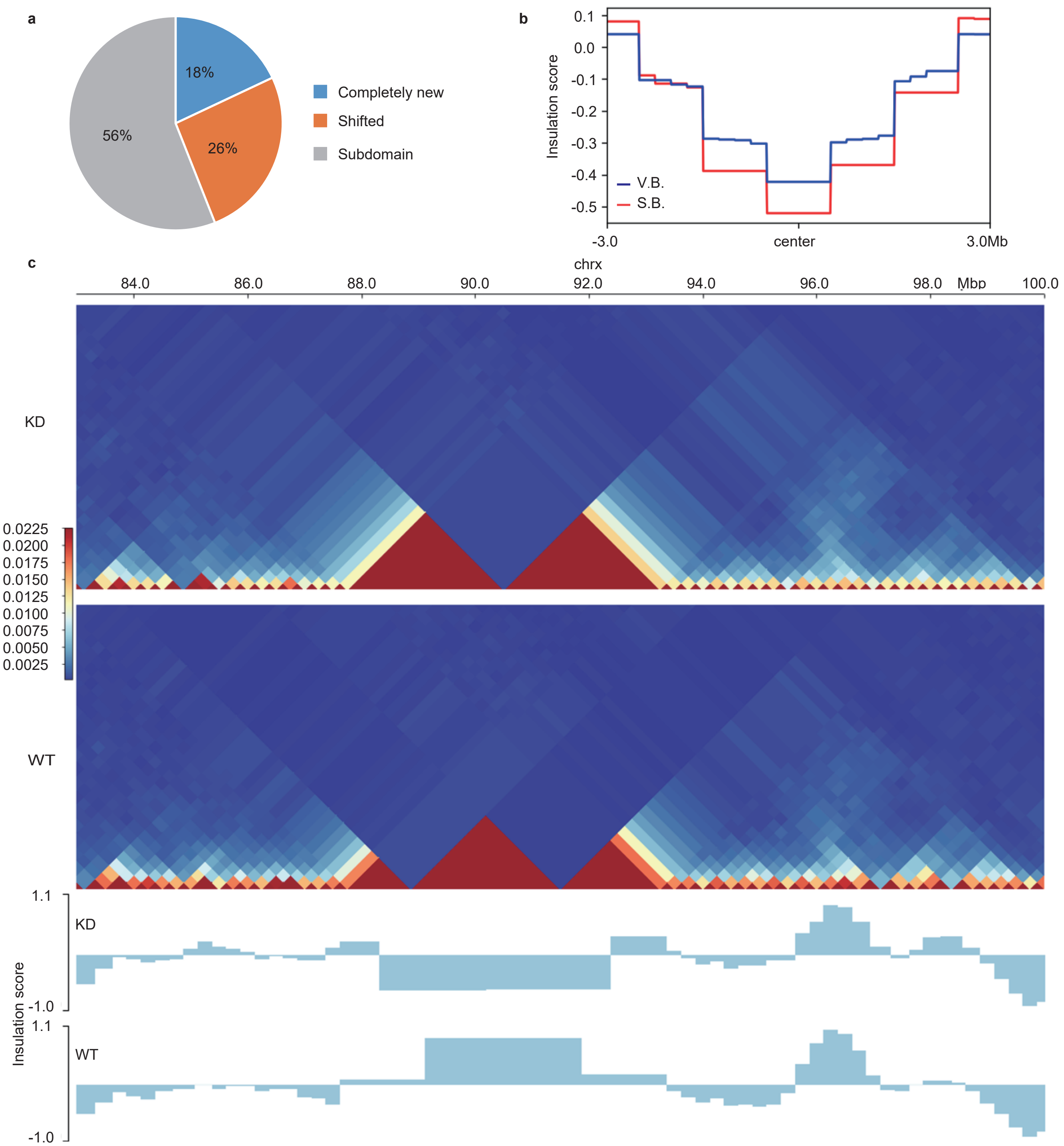
a
18%
26%
56%
Completely new
Shifted
Subdomain
b
Insulation score
0.1
0.0
-0.1
-0.2
-0.3
-0.4
-0.5
V.B.
S.B.
-3.0
center
3.0Mb
c
chrx
84.0
86.0
88.0
90.0
92.0
94.0
96.0
98.0
Mbp
100.0
KD
WT
0.0225
0.0200
0.0175
0.0150
0.0125
0.0100
0.0075
0.0050
0.0025
Insulation score
1.1
-1.0
KD
1.1
-1.0
WT

Supplementary Figure 5. Insulation score across TAD boundaries. a Proportion of different types of TAD boundary changes upon STAG2 knockdown (KD). **b** Insulation profiles at 1 Mb resolution between variable and stable boundaries upon STAG2 KD. **c** Representative heat map matrices for changes in TAD boundaries at 250 kb resolution with insulation scores shown below.

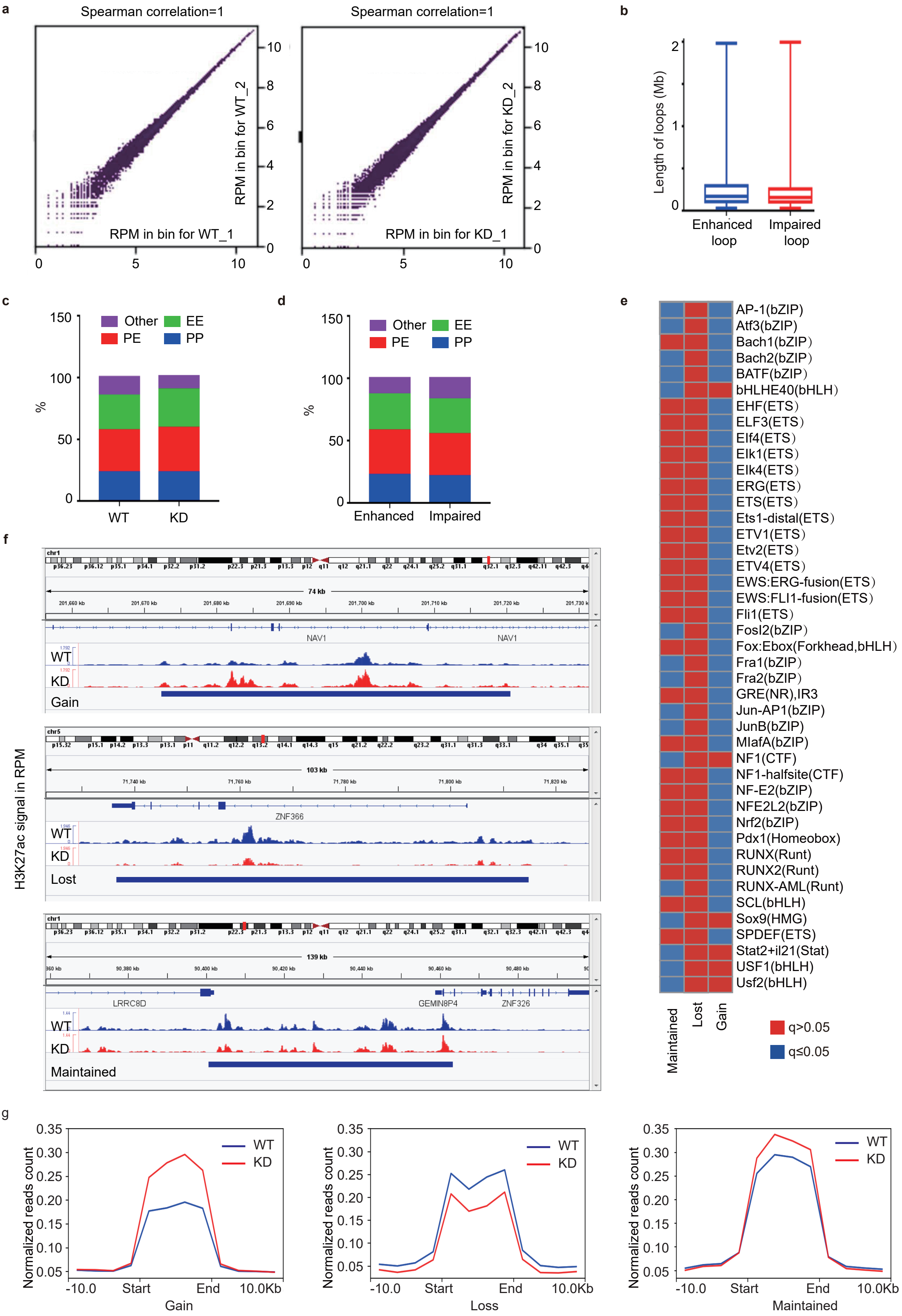
a
Spearman correlation=1
RPM in bin for WT_2
RPM in bin for WT_1
Spearman correlation=1
RPM in bin for KD_2
RPM in bin for KD_1
0
5
10
2
4
6
8
10
b
Length of loops (Mb)
0
1
2
Enhanced loop
Impaired loop
c
Other
EE
PE
PP
%
0
50
100
150
WT
KD
d
Other
EE
PE
PP
%
0
50
100
150
Enhanced
Impaired
e
AP-1(bZIP)
Atf3(bZIP)
Bach1(bZIP)
Bach2(bZIP)
BATF(bZIP)
bHLHE40(bHLH)
EHF(ETS)
ELF3(ETS)
Elf4(ETS)
Elk1(ETS)
Elk4(ETS)
ERG(ETS)
ETS(ETS)
Ets1-distal(ETS)
ETV1(ETS)
Etv2(ETS)
ETV4(ETS)
EWS:ERG-fusion(ETS)
EWS:FLI1-fusion(ETS)
Fli1(ETS)
Fosl2(bZIP)
Fox:Ebox(Forkhead,bHLH)
Fra1(bZIP)
Fra2(bZIP)
GRE(NR),IR3
Jun-AP1(bZIP)
JunB(bZIP)
MIafA(bZIP)
NF1(CTF)
NF1-halfsite(CTF)
NF-E2(bZIP)
NFE2L2(bZIP)
Nrf2(bZIP)
Pdx1(Homeobox)
RUNX(Runt)
RUNX2(Runt)
RUNX-AML(Runt)
SCL(bHLH)
Sox9(HMG)
SPDEF(ETS)
Stat2+il21(Stat)
USF1(bHLH)
Usf2(bHLH)
Maintained
Lost
Gain
q>0.05
q≤0.05
f
H3K27ac signal in RPM
chr1
74 kb
NAV1
WT
KD
Gain
chr5
103 kb
ZNF366
WT
KD
Lost
chr1
139 kb
LRRC8D
GEMIN8P4
ZNF326
WT
KD
Maintained
g
Normalized reads count
0.05
0.10
0.15
0.20
0.25
0.30
0.35
WT
KD
-10.0
Start
End
10.0Kb
Gain
Loss
Maintained

Supplementary Figure 6. Analyses of H3K27ac associated DNA loops and super-enhancers in M14 cells. a High Spearman's correlation of the reads distribution from H3K27ac HiChIP experiments is observed between two biological replicates of M14 cells without (WT) or with (KD) STAG2 knockdown. Spearman correlation is calculated with reads per million (RPM) in each 10Kb window throughout the whole genome. **b** Box plot of loop length for enhanced (n= 9115 loops) and impaired loops (n= 11327 loops) upon STAG2 knockdown. The box plot is defined by bounds at the 25th percentile and 75th percentile, centre at 50th percentile, the minima and maxima are at the 10th percentile and 90th percentile. **c** Percentages of four groups of H3K27ac associated DNA loops in M14 WT and KD cells. PE represents promoter-enhancer interaction; EE represents enhancer-enhancer interaction; PP represents promoter-promoter interaction; other represents loops with either anchor located outside of enhancer or promoter. An enhancer is defined by a H3K27ac peak of which the genomic location is outside of a promoter. A promoter is defined by the 1kb upstream of a transcriptional start site. **d** Percentage of the same four groups of interactions within enhanced or impaired H3K27ac associated DNA loops upon STAG2 knockdown. **e** Motifs enriched at gain, lost and maintained super-enhancers upon STAG2 KD in M14 cells. **f** Examples of gain, lost and maintained super-enhancers in M14 WT and KD cells. **g** Metaplot of H3K27AC signals across three types of super enhancers upon STAG2 KD.

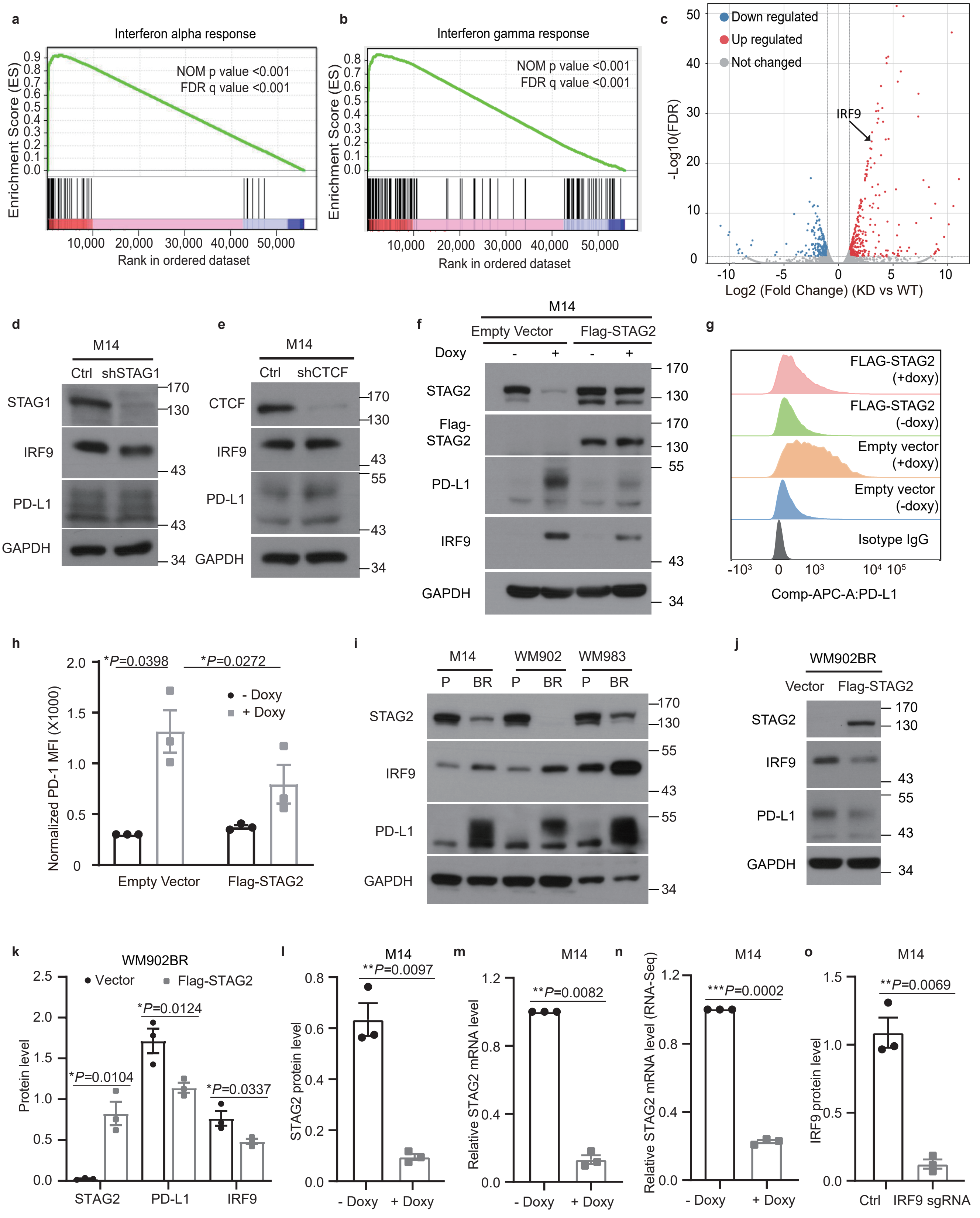
a
Interferon alpha response
NOM p value <0.001
FDR q value <0.001
Enrichment Score (ES)
Rank in ordered dataset
b
Interferon gamma response
NOM p value <0.001
FDR q value <0.001
Enrichment Score (ES)
Rank in ordered dataset
c
Down regulated
Up regulated
Not changed
IRF9
-Log10(FDR)
Log2 (Fold Change) (KD vs WT)
d
M14
Ctrl shSTAG1
STAG1
IRF9
PD-L1
GAPDH
e
M14
Ctrl shCTCF
CTCF
IRF9
PD-L1
GAPDH
f
M14
Empty Vector
Flag-STAG2
Doxy
STAG2
Flag-STAG2
PD-L1
IRF9
GAPDH
g
FLAG-STAG2 (+doxy)
FLAG-STAG2 (-doxy)
Empty vector (+doxy)
Empty vector (-doxy)
Isotype IgG
Comp-APC-A:PD-L1
h
*P=0.0398
*P=0.0272
- Doxy
+ Doxy
Normalized PD-1 MFI (X1000)
Empty Vector
Flag-STAG2
i
M14
WM902
WM983
P BR P BR P BR
STAG2
IRF9
PD-L1
GAPDH
j
WM902BR
Vector Flag-STAG2
STAG2
IRF9
PD-L1
GAPDH
k
WM902BR
Vector
Flag-STAG2
*P=0.0104
*P=0.0124
*P=0.0337
Protein level
STAG2
PD-L1
IRF9
l
M14
**P=0.0097
STAG2 protein level
- Doxy
+ Doxy
m
M14
**P=0.0082
Relative STAG2 mRNA level
- Doxy
+ Doxy
n
M14
***P=0.0002
Relative STAG2 mRNA level (RNA-Seq)
- Doxy
+ Doxy
o
M14
**P=0.0069
IRF9 protein level
Ctrl
IRF9 sgRNA

Supplementary Figure 7. Regulation of IRF9 and PD-L1 expression by STAG2 in melanoma cells. **a-b** GSEA enrichment plots depicting the enrichment of IFNα- (p=2.117e-25, q=2.367e-23) (**a**) and IFNγ response (p=2.341e-25, q=2.911e-23) (**b**) gene sets in M14 STAG2 KD cells. P value is the probability under the null distribution of obtaining an ES value that is as strong or stronger than that observed for the experiment under the permutation-generated null distribution. Q value is corrected by multiple comparisons at a false discovery rate (FDR) of 0.25. **c** Volcano plot of differentially expressed genes from RNA-seq data, with IRF9 indicated by the arrow. **d-e** M14 cells were transfected with STAG1 (**d**) or CTCF (**e**) shRNAs before subjected for immunoblotting with indicated antibodies. **f-h** Expression of a shRNA-refractory mutant of STAG2 rescued the effects of STAG2 knockdown on IRF9 and PD-L1 expression in M14 melanoma cells. Immunoblotting analyses (**f**) and flow cytometry analyses (**g,h**) were performed as indicated. **i** Immunoblotting analyses of STAG2, IRF9 and PD-L1 in several parental (P) and BRAF inhibitor-resistant (BR) melanoma cell lines. **j** Ectopic expression of STAG2 in WM902 BR cells increased the protein levels of IRF9 and PD-L1. **k** Quantification analyses (normalized to GAPDH) of data in **j.** **l-o** Quantifications of STAG2 knockdown by Western blot (**l**), RT-PCR (**m**) and RNA-seq (**n**) analyses, and IRF9 CRISPR knockout by Western blot analysis (**o**) in M14 cells. Data in **d-f,i-j** are representative of three independent experiments with similar results. Data in **h, k-o** are presented as mean ± SEM. *P* values were determined by paired two-tailed *t*-tests, $^*P < 0.05$; $^{**}P < 0.01$; $^{***}P < 0.001$.

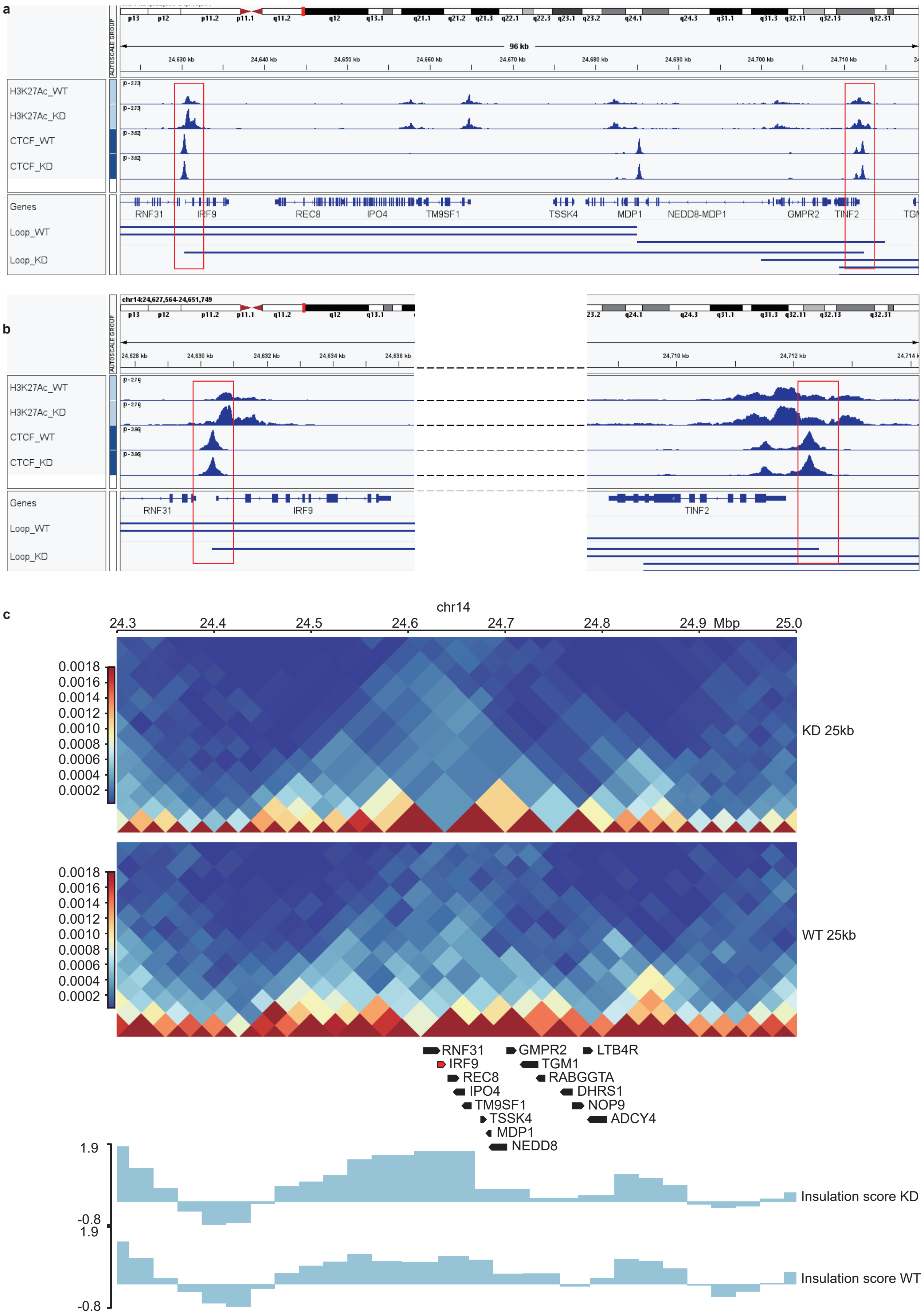

a
H3K27Ac_WT
H3K27Ac_KD
CTCF_WT
CTCF_KD
Genes
Loop_WT
Loop_KD
96 kb
RNF31
IRF9
REC8
IPO4
TM9SF1
TSSK4
MDP1
NEDD8-MDP1
GMPR2
TINF2
b
chr14:24,627,564-24,651,749
H3K27Ac_WT
H3K27Ac_KD
CTCF_WT
CTCF_KD
Genes
Loop_WT
Loop_KD
RNF31
IRF9
TINF2
c
chr14
24.3
24.4
24.5
24.6
24.7
24.8
24.9 Mbp
25.0
KD 25kb
WT 25kb
0.0018
0.0016
0.0014
0.0012
0.0010
0.0008
0.0006
0.0004
0.0002
RNF31
IRF9
REC8
IPO4
TM9SF1
TSSK4
MDP1
NEDD8
GMPR2
TGM1
RABGGTA
DHRS1
NOP9
ADCY4
LTB4R
1.9
-0.8
Insulation score KD
Insulation score WT

Supplementary Figure 8. Hi-C signal for the expanded TAD involving IRF9 regulation. a-b Location of anchors for a gain of chromatin interaction upon STAG2 knockdown (KD) in M14 cells**.** Left and right anchors are located around CTCF binding sites and overlapped with H3K27AC peak regions. **c** Representative contact frequency heatmap at 100 kb resolution showing an expanded TAD boundary from the right side of IRF9, leading to a new interaction formed between IRF9 and GMPR2 loci identified by HiChIP upon STAG2 KD.

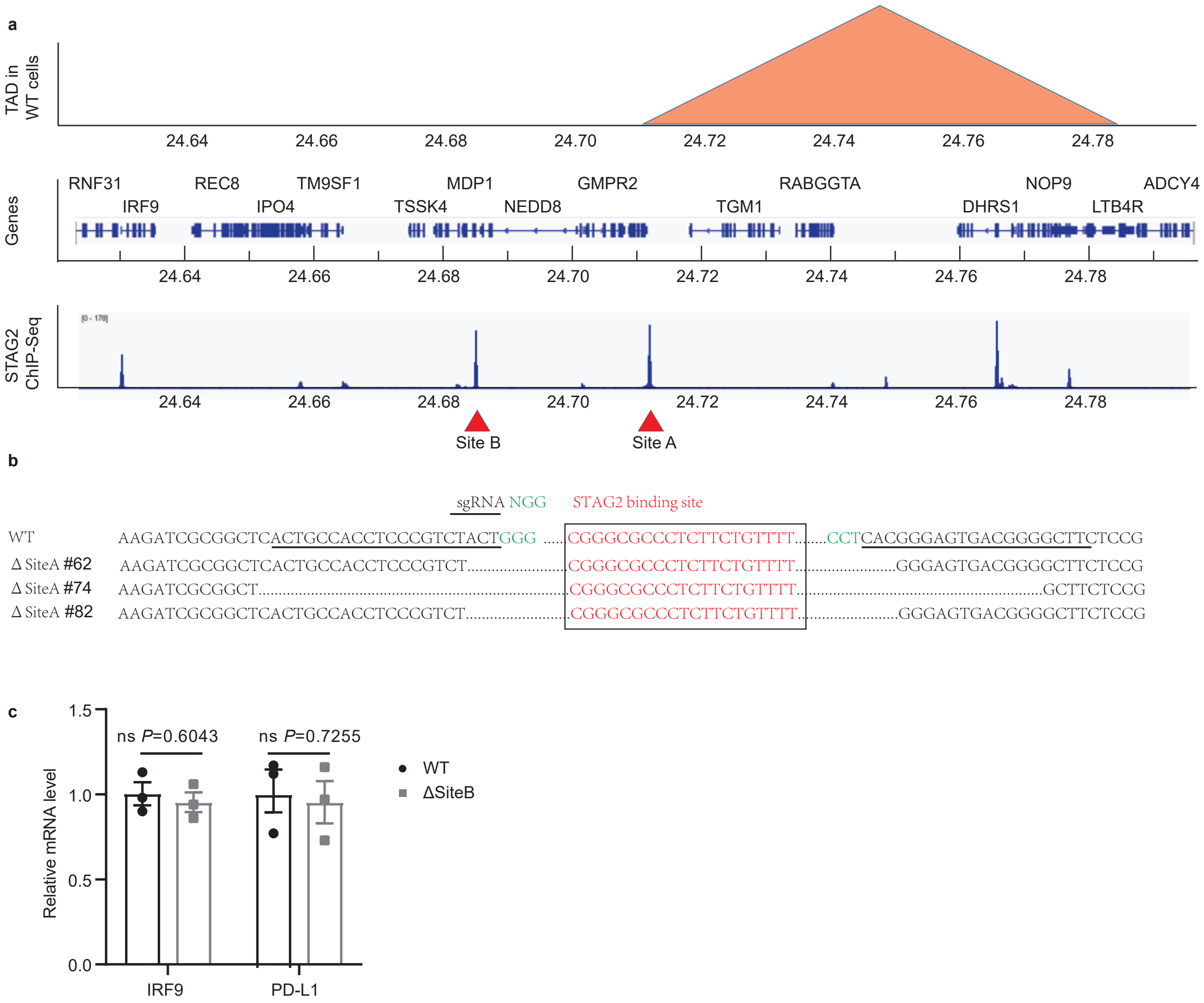
a
TAD in WT cells
24.64
24.66
24.68
24.70
24.72
24.74
24.76
24.78
Genes
RNF31
REC8
TM9SF1
MDP1
GMPR2
RABGGTA
NOP9
ADCY4
IRF9
IPO4
TSSK4
NEDD8
TGM1
DHRS1
LTB4R
STAG2 ChIP-Seq
Site B
Site A
b
sgRNA NGG
STAG2 binding site
WT AAGATCGCGGCTCACTGCCACCTCCCGTCTACTGGG ......CGGGCGCCCTCTTCTGTTTT.........CCTCACGGGAGTGACGGGGCTTCTCCG
Δ SiteA #62 AAGATCGCGGCTCACTGCCACCTCCCGTCT.........................CGGGCGCCCTCTTCTGTTTT.......................GGGAGTGACGGGGCTTCTCCG
Δ SiteA #74 AAGATCGCGGCT...CGGGCGCCCTCTTCTGTTTT...GCTTCTCCG
Δ SiteA #82 AAGATCGCGGCTCACTGCCACCTCCCGTCT.........................CGGGCGCCCTCTTCTGTTTT.......................GGGAGTGACGGGGCTTCTCCG
c
Relative mRNA level
1.5
1.0
0.5
0.0
ns P=0.6043
ns P=0.7255
IRF9
PD-L1
WT
ΔSiteB

Supplementary Figure 9. CRISPR/Cas9-mediated deletion of the STAG2 binding sites in the IRF9 region. **a** Schematic diagram indicates the positions of two STAG2 binding sites in the IRF9 region. **b** Sequencing results for M14 cell clones with CRISPR-engineered deletion of Site A or wild-type (WT) control. **c** Effects of CRISPR-engineered deletion of Site B on the mRNA expression levels of IRF9 and PD-L1 (n=3). Data in **c** are presented as mean ± SEM. *P* values were determined by unpaired two-tailed t-tests. ns $P > 0.05$.

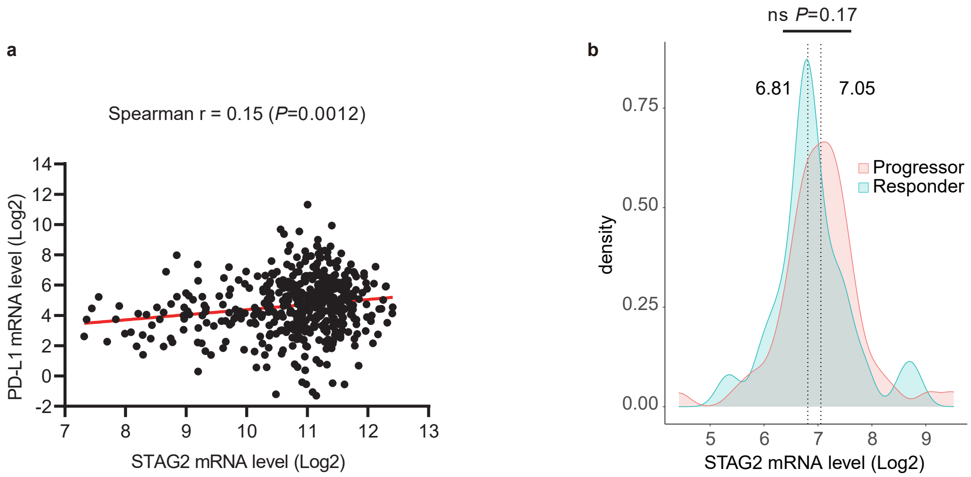
a
Spearman r = 0.15 (P=0.0012)
PD-L1 mRNA level (Log2)
STAG2 mRNA level (Log2)
b
ns P=0.17
6.81
7.05
Progressor
Responder
density
STAG2 mRNA level (Log2)

Supplementary Figure 10. Expression levels of STAG2 and PD-L1 in human melanoma samples. a Relationship between STAG2 mRNA level and PD-L1 mRNA level in TCGA SKCM patients (*n*=473) in the unadjusted analysis. The Spearman's correlation coefficient R value and two-sided P value are shown. **b** Median STAG2 mRNA expression level is higher in progressors (median = 7.05) compared to responders (median = 6.81) in a cohort of PD-1 ICB treated metastatic melanoma patients (Schadendorf cohort, *n* = 103 patients, 47 responders (CR/PR as best radiographic response to treatment) and 56 progressors (PD as best radiographic response to treatment), but the difference is not statistically significant (two-tailed Mann–Whitney–Wilcoxon (MWW), *P* = 0.17).

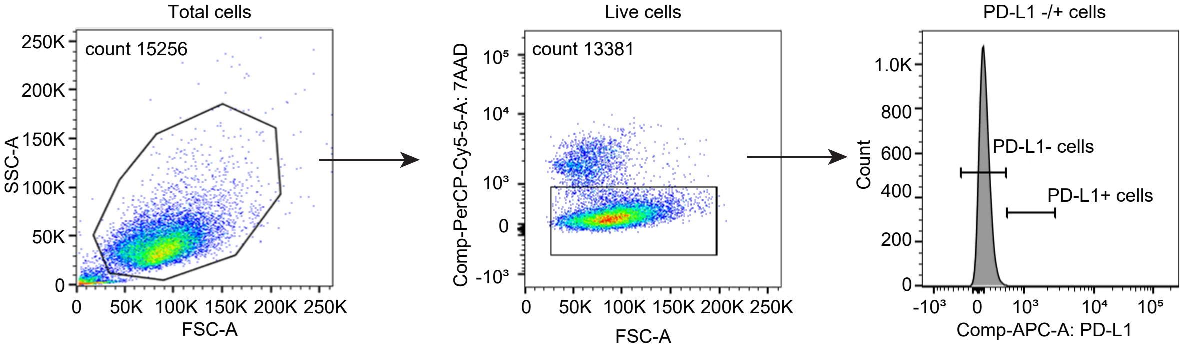

Total cells
count 15256
SSC-A
FSC-A
0
50K
100K
150K
200K
250K
Live cells
count 13381
Comp-PerCP-Cy5-5-A: 7AAD
FSC-A
10⁵
10⁴
10³
-10³
PD-L1 -/+ cells
Count
1.0K
800
600
400
200
PD-L1- cells
PD-L1+ cells
Comp-APC-A: PD-L1
-10³

Supplementary Figure 11. Gating strategy for flow cytometry analysis of PD-L1 surface expression. Live cells were identified by using SSC-A, FSC-A, and 7-AAD. PD-L1$^+$ cells were identified by using an isotype control.

Supplementary Table 1. Characterization of STAG1/2 switch and non-switch sites in the context of TAD domains, boundaries, promoters and enhancers.

Variable		Switch	Non-switch	P value	OR
Domain					
	In TADs	6350	11467	< 2.2e-16	0.6147439
	Not in TADs	7028	7802		
Boundary					
	Boundary	2220	2646	1.13e-12	1.24991
	Not Boundary	11158	16623		
Promoter					
	Promoter	581	859	0.622	0.9730338
	Non promoter	12797	18410		
Enhancer					
	Enhancer	71	636	< 2.2e-16	0.1563255
	Non enhancer	13307	18633		

The associations between switch/non-switch sites and 4 factors, including TAD domain, boundary, promoter and enhancer, were analyzed by two-sided fisher’s exact test.

Supplementary Table 2. Multivariate analysis of *PD-L1* expression.

			CD274			
Predictors	*Estimate*	*std. Error*	*CI*	*Statistic*	*P*	*df*
(Intercept)	-8.16	0.71	-9.55 – -6.77	-11.57	**2.14e-27**	470.00
STAT1	1.12	0.04	1.04 – 1.20	26.47	**3.43e-95**	470.00
STAG2	-0.13	0.06	-0.24 – -0.01	-2.17	**3.02e-02**	470.00
Observations	473					
R^2 / R^2 adjusted	0.609 / 0.608					

After adjusting for STAT1 expression, *STAG2* was an independent negative predictor of CD274 expression (β = - 0.13, *P* = 3.02e-02). *P* values were determined by two-sided alternative hypothesis test.
