# Supplementary material for: STAG2 regulates interferon signaling in melanoma via enhancer loop reprogramming
Source: Nat Commun. 2022 Apr 6;13:1859. doi: 10.1038/s41467-022-29541-9 (PMC8986786; doi:10.1038/s41467-022-29541-9)
Supplement: Supplementary file 3 — Description of additional Supplementary File [file 41467_2022_29541_MOESM3_ESM.pdf]

Description of additional supplementary Data files

Supplementary Data 1. Raw data quality control (QC) results across processing steps for Hi-C and HiChIP samples. The fraction of uniquely aligned read pairs was calculated based on the total number of initial reads. Self-circle and dangling-end fractions were calculated based on the total number of aligned read pairs. Intra- and inter-chromosomal contacts were calculated as a fraction of filtered valid interactions.
