# Supplementary material for: STAG2 regulates interferon signaling in melanoma via enhancer loop reprogramming
Source: Nat Commun. 2022 Apr 6;13:1859. doi: 10.1038/s41467-022-29541-9 (PMC8986786; doi:10.1038/s41467-022-29541-9)
Supplement: Supplementary file 5 — Reporting Summary [file 41467_2022_29541_MOESM5_ESM.pdf]

nature portfolio

Corresponding author(s): LEI GU
BIN ZHENG

Last updated by author(s): Feb 22, 2022

Reporting Summary

Nature Portfolio wishes to improve the reproducibility of the work that we publish. This form provides structure for consistency and transparency in reporting. For further information on Nature Portfolio policies, see our Editorial Policies and the Editorial Policy Checklist.

Statistics

For all statistical analyses, confirm that the following items are present in the figure legend, table legend, main text, or Methods section.

n/a	Confirmed	
☐	☒	The exact sample size (*n*) for each experimental group/condition, given as a discrete number and unit of measurement
☐	☒	A statement on whether measurements were taken from distinct samples or whether the same sample was measured repeatedly
☐	☒	The statistical test(s) used AND whether they are one- or two-sided<br>*Only common tests should be described solely by name; describe more complex techniques in the Methods section.*
☐	☒	A description of all covariates tested
☐	☒	A description of any assumptions or corrections, such as tests of normality and adjustment for multiple comparisons
☐	☒	A full description of the statistical parameters including central tendency (e.g. means) or other basic estimates (e.g. regression coefficient) AND variation (e.g. standard deviation) or associated estimates of uncertainty (e.g. confidence intervals)
☐	☒	For null hypothesis testing, the test statistic (e.g. *F*, *t*, *r*) with confidence intervals, effect sizes, degrees of freedom and *P* value noted<br>*Give P values as exact values whenever suitable.*
☒	☐	For Bayesian analysis, information on the choice of priors and Markov chain Monte Carlo settings
☒	☐	For hierarchical and complex designs, identification of the appropriate level for tests and full reporting of outcomes
☐	☒	Estimates of effect sizes (e.g. Cohen's *d*, Pearson's *r*), indicating how they were calculated

Our web collection on statistics for biologists contains articles on many of the points above.

Software and code

Policy information about availability of computer code

Data collection	Immunoblotting images were obtained using EPSON Scan .<br>Flow cytometry data were collected using BD FACSDiva (BD Biosciences, version 8.0.1).<br>RT-qPCR was performed on LightCycler 480 (Roche, Version 1.5).<br>NGS for ChIP-seq, RNA-seq, Hi-C were done with Illumina HiSeq 2500.<br>NGS for HiChIP was done with Illumina HiSeq 4000.
Data analysis	HiC and HiChIP paired-end reads were aligned to hg19 using HiC-Pro pipeline (VERSION 2.11.4) and visualized by the HiCPlotter (releases 7) and deeptools (3.0.2). QC was done by HOMER (4.11.1) “makeTagDirectory update” command. HOMER “analyzeHiC” command was used to generate and normalize contact matrices with 1Mb resolution. Chromatin compartment analysis (PCA) was applied using HOMER runHiCpca.pl and “analyzeHiC” command was then further used to generate DLR (Distal-to-Local [log2] Ratio) and ICF (Interchromosomal Fraction of Interactions) scores from Hi-C data. TADs were then identified by using findTADsAndLoops.pl and TADbit (v0.3). ChIP-seq reads were mapped to hg19 using bowtie2 (version 2.2.9). Low quality reads and PCR duplicates were filtered by samtools (version: 1.9) and picard toolkit (version: 2.8.0). Peaks were called by MACS2 (version: 2.1.1.20160309) against input controls. H3K27ac peaks was identified by hichip-peaks (version: 0.1.2) from the HiChIP data with FDR level at 0.001. H3K27ac-associated loops were identified using the FitHiChIP (Release 8.0). Differential loops were then identified by diffloop package in R (version 3.6.1). RNA-seq reads were mapped to the UCSC hg19 reference genome using STAR (version 2.7.0f) and the differential expression analysis was performed with rsem (version 1.3.0). RT-qPCR was analyzed using GraphPad prism (version 8.0.1).<br>Flow cytometry data were analysed using FlowJo (FlowJo, version 10.4).

For manuscripts utilizing custom algorithms or software that are central to the research but not yet described in published literature, software must be made available to editors and reviewers. We strongly encourage code deposition in a community repository (e.g. GitHub). See the Nature Portfolio guidelines for submitting code & software for further information.

Data

Policy information about availability of data

All manuscripts must include a data availability statement. This statement should provide the following information, where applicable:

- Accession codes, unique identifiers, or web links for publicly available datasets
- A description of any restrictions on data availability
- For clinical datasets or third party data, please ensure that the statement adheres to our policy

The sequencing data generated in this study have been deposited in the GEO database under accession code GSE156773

Field-specific reporting

Please select the one below that is the best fit for your research. If you are not sure, read the appropriate sections before making your selection.

[x] Life sciences   [ ] Behavioural & social sciences   [ ] Ecological, evolutionary & environmental sciences

For a reference copy of the document with all sections, see nature.com/documents/nr-reporting-summary-flat.pdf

Life sciences study design

All studies must disclose on these points even when the disclosure is negative.

Sample size	No sample size calculations were performed. The sample size was determined by the number of biological replicates required for ensuring statistical significance, and the sample sizes were chosen to support meaningful conclusions. The number of biological replicates is reported in the relevant figure legends in the manuscript.
Data exclusions	No data were excluded.
Replication	For each experiment, the number of biological independent sample is reported in the figure legend.
Randomization	Randomization is not relevant to this study since no in-vivo study was involved.
Blinding	Blinding is not relevant to this study since knowledge of the benchmark measure was essential to the analysis.

Reporting for specific materials, systems and methods

We require information from authors about some types of materials, experimental systems and methods used in many studies. Here, indicate whether each material, system or method listed is relevant to your study. If you are not sure if a list item applies to your research, read the appropriate section before selecting a response.

Materials & experimental systems

n/a	Involved in the study	
[ ]	[x]	Antibodies
[ ]	[x]	Eukaryotic cell lines
[x]	[ ]	Palaeontology and archaeology
[x]	[ ]	Animals and other organisms
[x]	[ ]	Human research participants
[x]	[ ]	Clinical data
[x]	[ ]	Dual use research of concern

Methods

n/a	Involved in the study	
[ ]	[x]	ChIP-seq
[ ]	[x]	Flow cytometry
[x]	[ ]	MRI-based neuroimaging

Antibodies

Antibodies used	Antibodies used for immunoblotting<br>Antibody (Vendor, catalog Number)<br>STAG2 (Santa Cruz, SC-81852)<br>IRF9 (Cell Signaling, #76684)<br>IRF7 (Cell Signaling, #4920)<br>USP18 (Cell Signaling, #4813)<br>ISG15 (Santa Cruz, SC-166755)<br>IRF1 (Cell Signaling, #8478)<br>IRF3 (Cell Signaling, #11904)<br>GAPDH (Cell Signaling, #2118)

GAPDH (Cell Signaling, #51332)
STAG1 (Novus Biologicals, NB100-298)
PD-L1 (R&D Systems, AF156)
CTCF (Cell Signaling, #2899)
Flag M2 (Sigma, F3165)

Antibodies used for flow cytometry
Antibody (Vendor, catalog Number)
APC anti-human PD-L1 antibody (Biolegend, 329708)
7-AAD viability staining solution (Biolegend, 420404)

Antibodies used for ChIP
Antibody (Vendor, catalog Number, lot number)
STAG1 (Abcam, ab4457, lot:GR279696-4)
STAG2 (Abcam, ab4464, Lot:GR271549-1)
SMC1A (Bethyl, A300-055A, Lot:5)
CTCF (Cell Signaling, 2899s, Lot:2)
H3K27ac (Abcam, ab4729)

Validation

All antibodies are commercially available and were validated as follows:

Antibodies used for immunoblotting
Antibody (Vendor, catalog Number, validation statements on the manufacturer's website showing it is suitable for WB in human)
STAG2 (Santa Cruz, SC-81852, https://www.scbt.com/zh/p/sa-2-antibody-j-12).
IRF9 (Cell Signaling, #76684, https://www.cellsignal.com/products/primary-antibodies/irf-9-d2t8m-rabbit-mab/76684).
IRF7 (Cell Signaling, #4920, https://www.cellsignal.com/products/primary-antibodies/irf-7-antibody/4920).
USP18 (Cell Signaling, #4813, https://www.cellsignal.com/products/primary-antibodies/usp18-d4e7-rabbit-mab/4813).
ISG15 (Santa Cruz, SC-166755, https://www.scbt.com/p/isg15-antibody-f-9?requestFrom=search).
IRF1 (Cell Signaling, #8478, https://www.cellsignal.com/products/primary-antibodies/irf-1-d5e4-xp-rabbit-mab/8478).
IRF3 (Cell Signaling, #11904, https://www.cellsignal.com/products/primary-antibodies/irf-3-d6i4c-xp-rabbit-mab/11904).
GAPDH (Cell Signaling, #2118, https://www.cellsignal.com/products/primary-antibodies/gapdh-14c10-rabbit-mab/2118).
GAPDH (Cell Signaling, #51332, https://www.cellsignal.com/products/antibody-conjugates/gapdh-d4c6r-mouse-mab-hrp-conjugate/51332).
STAG1 (Novus Biologicals, NB100-298, https://www.novusbio.com/products/sa1-antibody_nb100-298).
PD-L1 (R&D Systems, AF156, https://www.rndsystems.com/products/human-pd-l1-b7-h1-antibody_af156).
CTCF (Cell Signaling, #2899, https://www.cellsignal.com/products/primary-antibodies/ctcf-antibody/2899)
Flag M2 (Sigma, F3165, https://www.sigmaaldrich.com/US/en/product/sigma/f3165)

Antibodies used for flow cytometry
Antibody (Vendor, catalog Number, validation statements on the manufacturer's website showing its suitable for flow cytometry in human)
APC anti-human PD-L1 antibody (Biolegend, 329708, https://www.biolegend.com/en-us/products/apc-anti-human-cd274-b7-h1-pd-l1-antibody-4376)
7-AAD viability staining solution (Biolegend, 420404, https://www.biolegend.com/en-us/products/7-aad-viability-staining-solution-1649)

Antibodies used for ChIP
Antibody (Vendor, catalog Number, lot number). Validation information.
STAG1 (Abcam, ab4457, lot:GR279696-4). The manufacturer states that the antibody is suitable for ChIP in human (https://www.abcam.com/sa1-antibody-ab4457.html).
STAG2 (Abcam, ab4464, Lot:GR271549-1). The antibody has been used for ChIP in mouse previously (PMID: 22780989). The manufacturer stated that the antibody reacted with both human and mouse (https://www.abcam.com/sa2-antibody-ab4464.html).
SMC1A (Bethyl, A300-055A, Lot:5). The antibody has been used for ChIP in human previously (PMID: 32294452).
CTCF (Cell Signaling, 2899s, Lot:2). The manufacturer states that the antibody is suitable for ChIP in human (https://www.cellsignal.com/products/primary-antibodies/ctcf-antibody/2899).
H3K27ac (Abcam, ab4729). The manufacturer states that the antibody is suitable for ChIP in human (https://www.abcam.com/histone-h3-acetyl-k27-antibody-chip-grade-ab4729.html).

Eukaryotic cell lines

Policy information about cell lines

Cell line source(s)

Colo829 (Cat: #CRL-1974) and HEK293T(Cat: ACS-4500) were purchased from ATCC.
WM902, WM902-BR, WM983, WM983-BR, and MEL1617 cell lines were obtained from Dr. Meenhard Herlyn (Wistar Institute).
M14 and M14-BR were from Dr. David Fisher (Cutaneous Biology Research Center, MGH)

Authentication

All cell lines were authenticated by using STR fingerprinting.

Mycoplasma contamination

All cells lines were confirmed without mycoplasma contamination.

Commonly misidentified lines
(See ICLAC register)

No commonly misidentified lines were used in this study

ChIP-seq

Data deposition

☒ Confirm that both raw and final processed data have been deposited in a public database such as GEO.

☒ Confirm that you have deposited or provided access to graph files (e.g. BED files) for the called peaks.

Data access links
May remain private before publication.

GSE156773

Files in database submission

raw files:
CTCF_stag2kd.R1.fastq
CTCF_stag2wt.R1.fastq
input_stag2kd.R1.fastq
input_stag2wt.R1.fastq
SMC1A_stag2kd.R1.fastq
SMC1A_stag2wt.R1.fastq
STAG1_stag2kd__S2_L001_R1_001.fastq
STAG1_stag2kd_S2_L002_R1_001.fastq
STAG1_stag2kd_S2_L003_R1_001.fastq
STAG1_stag2kd_S2_L004_R1_001.fastq
STAG1_stag2wt_S1_L001_R1_001.fastq
STAG1_stag2wt__S1_L002_R1_001.fastq
STAG1_stag2wt__S1_L003_R1_001.fastq
STAG1_stag2wt__S1_L004_R1_001.fastq
STAG2_stag2kd_S4_L001_R1_001.fastq
STAG2_stag2kd_S4_L002_R1_001.fastq
STAG2_stag2kd_S4_L003_R1_001.fastq
STAG2_stag2kd_S4_L004_R1_001.fastq
STAG2_stag2wt_S3_L001_R1_001.fastq
STAG2_stag2wt_S3_L002_R1_001.fastq
STAG2_stag2wt_S3_L003_R1_001.fastq
STAG2_stag2wt_S3_L004_R1_001.fastq
new_SA1-M14-Ctl.fq.gz
new_SA1-M14-SA2-KD.fq.gz
new_SA2-M14-Ctl.fq.gz
new_SA2-M14-SA2-KD.fq.gz
new_CTCF-M14-Ctl.fq.gz
new_CTCF-M14-SA2-KD.fq.gz
new_Smc1-M14-Ctl.fq.gz
new_Smc1-M14-SA2-KD.fq.gz
new_Input-M14-Ctl.fq.gz
new_Input-M14-SA2-KD.fq.gz

peak files:
ctcf_stag2kd_peaks_m2q01.bed
ctcf_stag2wt_peaks_m2q01.bed
SMC1A_stag2kd_peaks_m2q01.bed
SMC1A_stag2wt_peaks_m2q01.bed
STAG1_stag2kd_peaks_m2q01.bed
STAG1_stag2wt_peaks_m2q01.bed
STAG2_stag2kd_peaks_m2q01.bed
STAG2_stag2wt_peaks_m2q01.bed
SA1_sa2wt_peaks.narrowPeak
SA1_sa2kd_peaks.narrowPeak
SA2_sa2wt_peaks.narrowPeak
SA2_sa2kd_peaks.narrowPeak
CTCF_sa2wt_peaks.narrowPeak
CTCF_sa2kd_peaks.narrowPeak
SMC1A_sa2wt_peaks.narrowPeak
SMC1A_sa2kd_peaks.narrowPeak

bigwig files:

SA1_sa2wt.bw
SA1_sa2kd.bw
SA2_sa2wt.bw
SA2_sa2kd.bw
CTCF_sa2wt.bw
CTCF_sa2kd.bw
SMC1A_sa2wt.bw
SMC1A_sa2kd.bw
Input_sa2wt.bw
Input_sa2kd.bw

Genome browser session (e.g. UCSC)	hg19

Methodology

Replicates	2
Sequencing depth	>20 million reads
Antibodies	The antibodies used included:<br>STAG1 (Abcam, ab4457, lot: GR279696-4)<br>STAG2 (Abcam, ab4464, Lot: GR271549-1)<br>SMC1A (Bethyl, A300-055A, lot: 5)<br>CTCF (Cell Signaling Tech, 2899s, lot: 2).
Peak calling parameters	Sequencing reads were aligned to the human hg19 genome using bowtie2 (version 2.2.9). macs2 (version 2.1.1.20160309) callpeak -g hs -q 0.05 -t replicate1.bam -c input1.bam
Data quality	fastq files were quality (Phred Score 20) and adapter trimmed using bbmap. Sequence enrichment were checked using cummulative sums of the reads count of binned genome. All the peaks were filtered for 5%FDR. The number of peaks with 5%FDR and fold enrichment >5.
Software	Software: MACS2 peak caller was used to find significant peak signals for each sample above background of the input.

Flow Cytometry

Plots

Confirm that:

- [x] The axis labels state the marker and fluorochrome used (e.g. CD4-FITC).
- [x] The axis scales are clearly visible. Include numbers along axes only for bottom left plot of group (a 'group' is an analysis of identical markers).
- [x] All plots are contour plots with outliers or pseudocolor plots.
- [x] A numerical value for number of cells or percentage (with statistics) is provided.

Methodology

Sample preparation	Cells were collected, washed and resuspended in PBS containing 10% FBS. Single-cell suspensions were stained at a 1:20 dilution with APC anti-human PD-L1 antibody (Biolegend, 329708) or isotype control (Biolegend, 400319) for 30 minutes on ice. Cells were then washed and resuspended in 300ul PBS, add 3ul 7AAD (Biolegend, 420404) into the samples before running them on a flow cytometer.
Instrument	BD FACSCanto™ II
Software	FACSDiva software (BD Biosciences) was used to collect the data, and the data was analyzed by FlowJo software (FlowJo, LLC, version 10.4).
Cell population abundance	At least 20000 cells were acquired for each sample
Gating strategy	Live cells were identified by using SSC-A, FSC-A, and 7-AAD. PD-L1+ cells were identified by using an isotype control.

- [x] Tick this box to confirm that a figure exemplifying the gating strategy is provided in the Supplementary Information.
